# Supplementary material for: In Vivo Evaluation of Fibroblast Growth Factor Receptor Inhibition in Mouse Xenograft Models of Gastrointestinal Stromal Tumor
Source: Biomedicines. 2022 May 13;10(5):1135. doi: 10.3390/biomedicines10051135 (PMC9138864; doi:10.3390/biomedicines10051135)
Supplement: Supplementary file 1 [file biomedicines-10-01135-s001.zip › Schoffski et al_Biomedicines_Supplementary Table S1.pdf]

Supplementary Table S1: Animals lost during experiment.

**Model/passage**	**Treatment group**	**Day of experiment**	**Remark**
UZLX-GIST9p.4	Imatinib	7	Tumor exceeding acceptable volume
	Imatinib	10	Body weight loss (<80% of baseline)
	Dovitinib	8	Abnormal tumor growth (into peritoneal cavity)
	Dovitinib	21	Found dead, body weight loss (>80%)
UZLX-GIST2p.17	Binimetinib	13	Body weight loss (<80% of baseline)
	Binimetinib	20	Body weight loss (<80% of baseline)
GIST48	Control	3	Body weight loss (<80% of baseline)
	Imatinib	13	Body weight loss (<80% of baseline)
