# Supplementary figures and images for: In Vivo Evaluation of Fibroblast Growth Factor Receptor Inhibition in Mouse Xenograft Models of Gastrointestinal Stromal Tumor
Source: Biomedicines. 2022 May 13;10(5):1135. doi: 10.3390/biomedicines10051135 (PMC9138864; doi:10.3390/biomedicines10051135)

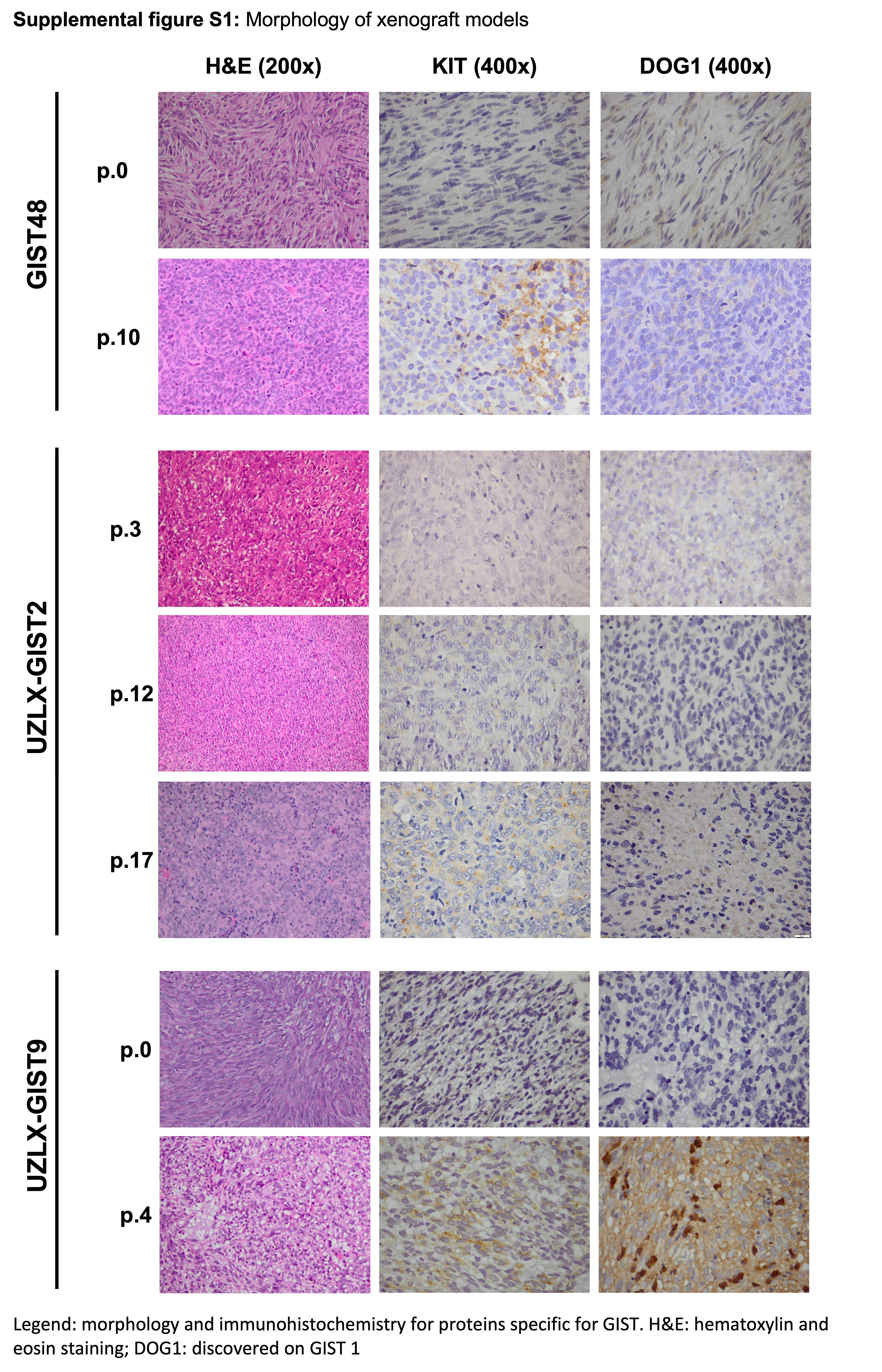

Supplement: Supplementary file 1 [file biomedicines-10-01135-s001.zip › Schoffski et al_Biomedicines_Figure S1_legend.png]

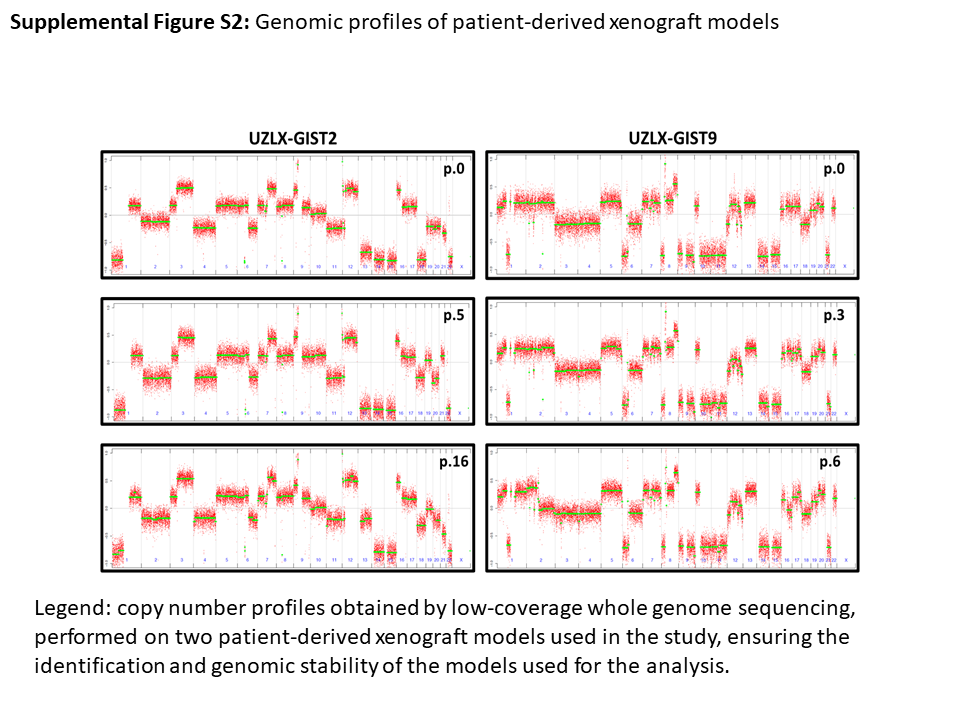

Supplement: Supplementary file 1 [file biomedicines-10-01135-s001.zip › Schoffski et al_Biomedicines_Figure S2_legend.png]

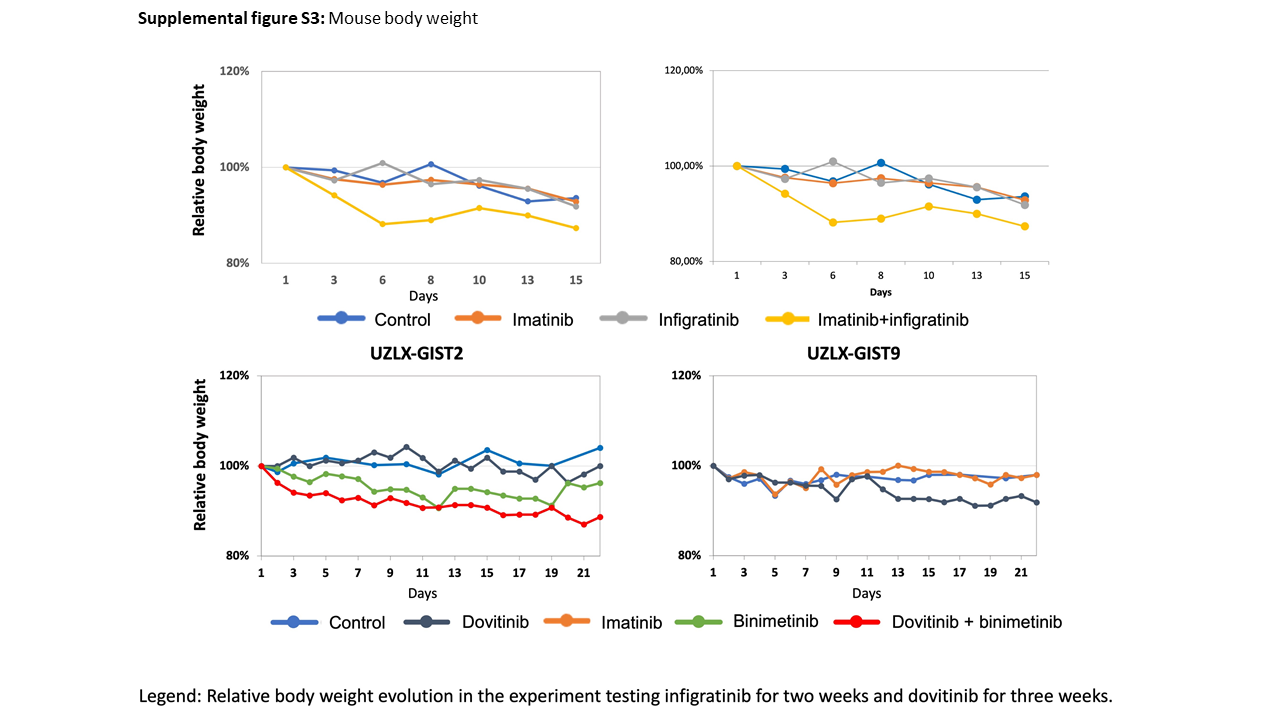

Supplement: Supplementary file 1 [file biomedicines-10-01135-s001.zip › Schoffski et al_Biomedicines_Figure S3_legend.png]
